# Skin biomechanics: a potential therapeutic intervention target to reduce scarring

**DOI:** 10.1093/burnst/tkac036

**Published:** 2022-08-23

**Authors:** Motaharesadat Hosseini, Jason Brown, Kiarash Khosrotehrani, Ardeshir Bayat, Abbas Shafiee

**Affiliations:** Centre for Biomedical Technologies, School of Mechanical, Medical and Process Engineering (MMPE), Faculty of Engineering, Queensland University of Technology, Brisbane, QLD 4059, Australia; Herston Biofabrication Institute, Metro North Hospital and Health Service, Brisbane, QLD 4029, Australia; Royal Brisbane and Women's Hospital, Metro North Hospital and Health Service, Brisbane, QLD 4029, Australia; The University of Queensland Diamantina Institute, Translational Research Institute, The University of Queensland, Brisbane, QLD 4102, Australia; Centre for Dermatology Research, NIHR Manchester Biomedical Research Centre, Stopford Building, University of Manchester, Oxford Road, Manchester, M13 9PT, England, UK; MRC-SA Wound Healing Unit, Hair & Skin Research Laboratory, Division of Dermatology, University of Cape Town, Cape Town 7935, South Africa; Herston Biofabrication Institute, Metro North Hospital and Health Service, Brisbane, QLD 4029, Australia; Royal Brisbane and Women's Hospital, Metro North Hospital and Health Service, Brisbane, QLD 4029, Australia; The University of Queensland Diamantina Institute, Translational Research Institute, The University of Queensland, Brisbane, QLD 4102, Australia

**Keywords:** Dermal fibrosis, Mechanotransduction, Pressure therapy, Tension therapy, Wound healing, Skin biomechanics

## Abstract

Pathological scarring imposes a major clinical and social burden worldwide. Human cutaneous wounds are responsive to mechanical forces and convert mechanical cues to biochemical signals that eventually promote scarring. To understand the mechanotransduction pathways in cutaneous scarring and develop new mechanotherapy approaches to achieve optimal scarring, the current study highlights the mechanical behavior of unwounded and scarred skin as well as intra- and extracellular mechanisms behind keloid and hypertrophic scars. Additionally, the therapeutic interventions that promote optimal scar healing by mechanical means at the molecular, cellular or tissue level are extensively reviewed. The current literature highlights the significant role of fibroblasts in wound contraction and scar formation via differentiation into myofibroblasts. Thus, understanding myofibroblasts and their responses to mechanical loading allows the development of new scar therapeutics. A review of the current clinical and preclinical studies suggests that existing treatment strategies only reduce scarring on a small scale after wound closure and result in poor functional and aesthetic outcomes. Therefore, the perspective of mechanotherapies needs to consider the application of both mechanical forces and biochemical cues to achieve optimal scarring. Moreover, early intervention is critical in wound management; thus, mechanoregulation should be conducted during the healing process to avoid scar maturation. Future studies should either consider combining mechanical loading (pressure) therapies with tension offloading approaches for scar management or developing more effective early therapies based on contraction-blocking biomaterials for the prevention of pathological scarring.

## Graphical Abstract

**Graphical Abstract:**
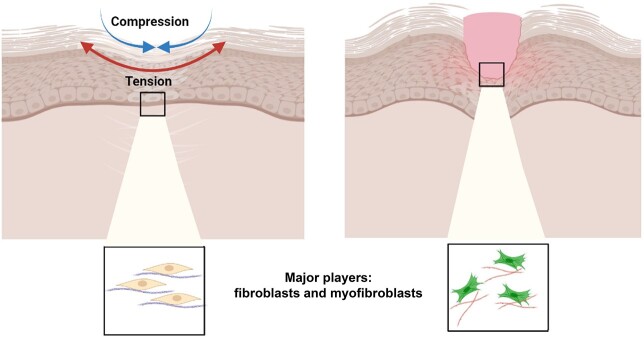

Highlights

- The contribution of key progenitor cells in skin fibrosis highlighted.
- The biomechanical characteristics of healthy and scarred skin tissues presented.
- The role of mechanical forces and mechanotransduction pathways in skin scarring described.
- The current and emerging therapeutic approaches to minimize scarring by modulating the mechanical forces presented.

## Background

Upon skin wounding, the activation and coordination of numerous intracellular and intercellular signaling are required to restore tissue integrity and homeostasis. Meanwhile, various immune cells, coagulation cascades and inflammatory processes are activated to promote the healing processes. Notable morphological alterations happen in different cell types, such as keratinocytes, immune cells, endothelial cells and dermal fibroblasts that ultimately culminate in cellular proliferation, differentiation and migration [1,2]. If all these reactions are successful and keep the organ functioning, repair ends up with a macroscopic fibrous disturbance, mostly containing fibroblasts and collagen, which slowly remodels into scar tissues [3]. Scarring, thus, is the clinical outcome of the normal wound healing process with characteristic events of inflammation, fibroplasia, granulation tissue formation and scar maturation.

After wounding, the blood inflammatory cells are recruited into the wound site. Then, the acute inflammatory responses are induced by residential macrophages as well as mast cells that lead to the activation of fibroblasts and endothelial cells, which are responsible for the release of tissue components (i.e. fibrillogenesis), as well as the formation of new blood vessels (i.e. angiogenesis), respectively. Meanwhile, the granulation tissue is formed and then reorganized. With the differentiation of fibroblasts into myofibroblasts, extracellular matrix (ECM) is deposited, and the wound size is decreased. Scar formation finally arises from the excessive accumulation of unorganized fibrous networks. In the adult human, scar remodeling may continue for months or even years, and the recovery of the normal ECM architecture is never accomplished completely [4,5].

In humans, wounds that have impairment in healing can be considered either as delayed wound healing, including venous or arterial ulcers, pressure sores and diabetic ulcers, or as excessive/hyperproliferative wound healing, including pyogenic granuloma, keloids and hypertrophic scars [6]. The present review aims to focus on excessive healing where large amounts of ECM are deposited, and local vascularization and cell proliferation are affected. The endpoint of overhealing is excessive fibrotic reactions evidenced by the deposition of large disfiguring fibrotic tissues and distortion in surface structures and dermal architecture.

Major injuries such as burns can lead to the development of abnormal raised scars such as hypertrophic scars. Keloids are raised tissues that spread beyond the original wound area and form when scar tissue grows excessively. The keloid scars may develop after minor skin damage, such as an acne spot [1]. Mechanical stress during wound repair can contribute to extended wound healing and excessive scar formation [7]. The suitable mechanical stresses can facilitate the survival of myofibroblast and trigger the expression of alpha-smooth muscle actin (α-SMA). In the scar microenvironment, there is evidence of prolonged inflammation and oxidative insults characterized by the excessive deposition of ECM and inhibition of cytoprotective enzyme heme oxygenase-1 [8].

The association between mechanical tension and skin scarring is not new, and the evidence from fetal mammalian skin with thin collagen fibers and low resting stress supports this concept [1]. Scarless fetal wound healing occurs in an inherently low resting tension environment, thus providing a clue in identifying influential molecules and cells. For example, fetal wounds may be healed with no scar due to no or a relatively low number of inflammatory cells [9], increased ratio of type III : I collagen [4], few or less mature mast cells [4], and low level of transforming growth factor (TGF)-β components [10]. It is still unclear how the changes in skin biomechanics contribute to scarring.

The current study intends to describe the mechanics of uninjured skin and cutaneous wounds. Also, the contribution of mechanical forces in scar development is reviewed, considering both intra- and extracellular mechanisms. The biomechanical behavior of skin fibroblast, keratinocytes and myofibroblast and the key mechanical signaling pathways in play are presented. Additionally, significant preclinical and clinical evidence where skin scarring is treated by advanced mechanotherapies is reviewed.

## Review

### Mechanical forces in cutaneous wound healing and scarring

Human skin is subjected to intrinsic and extrinsic mechanical forces [11]. Mechanical forces contribute to wound healing and increase the susceptibility of certain body parts to excessive cutaneous scarring [12,13]. Therefore, understanding the correlation between mechanical forces, wound healing and scarring is crucial.

### Mechanical characteristics of unwounded and scarred skin

**Figure 1.**
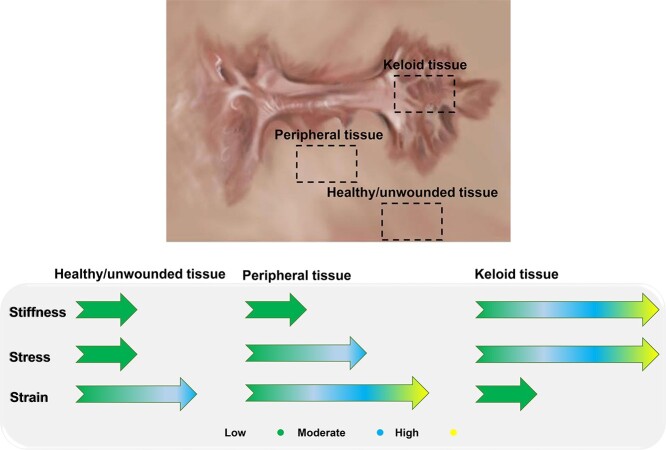
Complicated mechanical factors mediate scar progression in keloids. The mechanical stress is the highest in the keloidal tissues, followed by peripheral tissues. The normal unwounded skin represents the lowest mechanical stress. The keloid peripheral tissues and keloidal tissues demonstrate the highest and lowest mechanical strains, respectively. Source: Reprinted with permission from [28]. Open access; SpringerLink.

The skin is the body’s outmost layer. As such, the skin requires strong pliability to maintain its elasticity, extensibility, firmness and tensile strength [14]. First, some terms should be described. Stress refers to force per area and can be considered a measure of force intensity. Strain shows the percentage of deformation or changes in length or shapes upon applied stress. The human body is typically exposed to mechanical stresses, including tension as positive stress and compression as negative stress (Figure 1). Skin responds to the intrinsic mechanical forces in both tissue and cellular levels, and the external tensile stress is known as tension [15]. Inherent skin tension is implicated in maintaining homeostasis as well as causing pathological scarring [16]. Skin tensile stress is considered positive stress, largely with an anisotropic behavior because of the nonlinear viscoelasticity in skin tissue [11,17]. This tension is caused by different sources; bones like the sternum or tubercles resemble strong anchors below the skin, which locally augment the skin tension [17]. External objects and jewelry such as earrings concentrate tension close to piercing sites [18]. The homeostasis of skin biomechanics and mechanobiology is dependent on the equilibrium of the forces exerted on the skin from the outside and inside of the body [16].

Nearly all skin cells actively interact with physical forces, and such a feature can define the response of uninjured and injured skins to the physical properties of their surroundings. Following sternotomy and skin defects around joints, for example, scar formation becomes worst due to exposure to high mechanical force [19,20]. On the contrary, pathologic scarring is reduced in cesarean section wounds after offloading of physical force [21]. When skin tension increases, the ECM and cells become elongated or expanded in shape, and, in turn, the stiffness and rigidity of the tissue increase as a whole. It has been shown that scarring elevates the skin stiffness to 3.0 N/mm compared to 0.75 N/mm in healthy skin [22,23]. In addition to tension, other factors such as compression, shear and osmotic forces are of great importance to improve skin wound healing [11,24,25]. It has been shown, for example, that incisions placed parallel to the Langer lines, which refer to the main tension lines in human skin, are associated with decreased tension and tend to develop thinner scars with less collagen generation and deposition during the healing process as opposed to those made perpendicular [19,26,27,28]. Unlike the skin eyelid, the back, sternum, joints, etc., as parts of the body exposed to high mechanical loads, are associated with increased amounts of scarring upon injury [19,20].

Scar tissue tends to be permanently weaker than normal skin. Even though the strength of wounded skin gradually increases during scar maturation, achieving 20% of its final strength within the first three weeks [2] and 50% by six weeks of healing [29], scar tissue will only ever reach 80% of the uninjured skin strength [30]. This difference in the mechanical behavior of normal skin and scar tissue partly arises from the lack of rete ridges and elastic fibers in scars [31,32]. Scars had considerably decreased failure properties, resulting in compromised bursting strength, extensibility and toughness when compared to unwounded skin [33,34]. The reduction in failure resistance would be indicative of less total collagen content in the scar tissue cross-section since its collagen fibers are either small in size or number. Though almost no effect on high load linear stiffness [33], scarring causes a more noticeable response for low load behavior. Due to significantly stiff responses at low loads, there is a greater strain in the scar collagen fibers, leading to a greater wringing-out effect in the tissue. Scars exhibit an increased viscous behavior that might be because of higher water content, or due to raised content of proteoglycan, expression of various proteoglycans, or a combination of these factors [35].

The local microenvironment and the way mechanical forces are distributed are important factors guiding scar development. In mice, a hypertrophic scar is formed if stretching is applied to a full-thickness surgical wound [7]. This type of scar is found to show less extensibility, needs more strain energy during physiological stretching, and fails to store the energy more efficiently as compared to uninjured healthy skin [36]. These observations might arise from the composition and structure of unwounded and scarred skin tissues. Hypertrophic scarring is likely to have a partially pre-aligned collagen matrix with a tendon-like crimping pattern, which decreases the extensibility of this scarred tissue and increases the strain energy required to stretch it. Moreover, the absence of a complete elastic fiber network, a normal glycosaminoglycan content, and/or functional collagen fiber slippage accounts for the reduction in the ability of hypertrophic scar to return strain energy [36]. Despite more stiffness observed in this scar than in normal skin [37], the maximum stiffness of both skin types is the same. Thus, the apparent elevation in the rigidity of hypertrophic scar may result from reduced extensibility rather than altered stiffness [36].

Keloidal scars tend to be found on the anterior chest and upper back exposed to mechanical load [38]. Furthermore, they progress in certain shapes, including butterflies or dumbbells, highlighting the role of mechanotransduction dysregulation in the formation of keloids [39,40]. In contrast to the surrounding unwounded skin, keloids show different and complex mechanical behaviors in response to stress and strain [41]. By comparing the anatomical sites with high to almost no incidence of keloid development, Dohi et al. figured out how these sites react to the mechanical loading. The amount of mechanical stress is highest in keloidal tissues, followed by peripheral tissue close to these scars and then unwounded healthy skin. Further analysis indicated that the elevation of strain in the peripheral tissues and the keloid’s proximity may trigger mechanotransduction pathways that eventually mediate keloid progression **(**Figure 1**)** [41].

### Biomechanical behavior of cellular skins

The ECM and extracellular fluid transduce mechanical cues into the cells and regulate the cutaneous remodeling. The cell membrane and cytoskeletal components (e.g. actin), ion channels, catenin complexes, cell adhesion molecules (e.g. integrins), and several signaling pathways (e.g. Wnt) have been reported as mechanosensors [42–44]. These molecules allow the transmission of mechanical signals into the cells, which bind to the ECM and induce a variety of biological responses [45–47]. In the wounded skin, the mechanosensors are not fully functional and cannot properly respond to the mechanical stimuli [48].

Mechanical forces carry impacts on cellular behaviors, such as gene expression, proliferation and migration [49–53]. Such physical cues from the extracellular microenvironment can change mechanosensors (e.g. ROCK), which affect gene expression in the long term [54–60]. The use of mechanical forces in mice during the early phases of wound healing causes a reduction of protein kinase B (Akt)-dependent apoptosis and produces hypertrophic scarring [7]. Injuries like trauma, burn, etc., make the skin susceptible to cutaneous disorders, including fibrogenesis or keratinocyte hyperproliferation. In the following, the responses of skin key cells in cutaneous scarring, as well as their responses to mechanical stresses, are highlighted. These reactions, however, are not limited to cell activities only but also extended to gene and protein interactions, which, in our opinion, can point to therapeutic targets for preventing scars and restoring skin regeneration.

### Skin fibroblast and mechanisms involved in mechanical forces-mediated scar remodeling

Although ECM deposition and temporary scar formation constitute the process of normal healing, fibrotic scars involve abnormal ECM reconstruction with structural stiffness. The mechanical microenvironment has effects on scar contracture. When contraction still occurs after healing, the outcome will be tissue with poor functional and cosmetic features [12,61–63].

Fibroblasts, as major contributors to the wound healing process, could be influenced by exogenous mechanical forces, which can elevate the expression of fibrotic genes, such as TGF-β, α-smooth muscle actin (α-SMA), and collagen I. In this response, a variety of mechanoreceptors, such as integrins, growth factor receptors, G protein-coupled receptors, and ion channels, are involved [12,64]. Fibroblasts are heterogeneous populations and composed of several subpopulations with definite roles [13,14,25,29]. Wound injuries stimulate a fibroblast subset in the dermis leading to contraction, ECM production and eventually fibrotic scarring [33–35]. Rinkevich et al. found a dermal fibroblast subpopulation characterized by the embryonic gene expression of *Engrailed-1* (*En1* lineage–positive fibroblasts) responsible for dorsal skin scars [65]. Recently, Mascharak et al. reported that *En1* lineage–negative fibroblasts act on the *En1* gene in mechanical stimuli-exposed wounds and cause scar formation as postnatal *En1* lineage–positive fibroblasts. The direct or indirect knockdown of *En1* reduces mechanically induced fibrogenic changes, thereby enhancing skin regeneration by *En1* lineage–negative fibroblasts. Thus, *En1* itself is a mechanoresponsive master regulator activating fibroblasts. Residual *En1* lineage–negative fibroblasts in postnatal mammalian skin are capable of skin regeneration when the mechanically driven preference for fibrosis can be inhibited [66].

**Figure 2.**
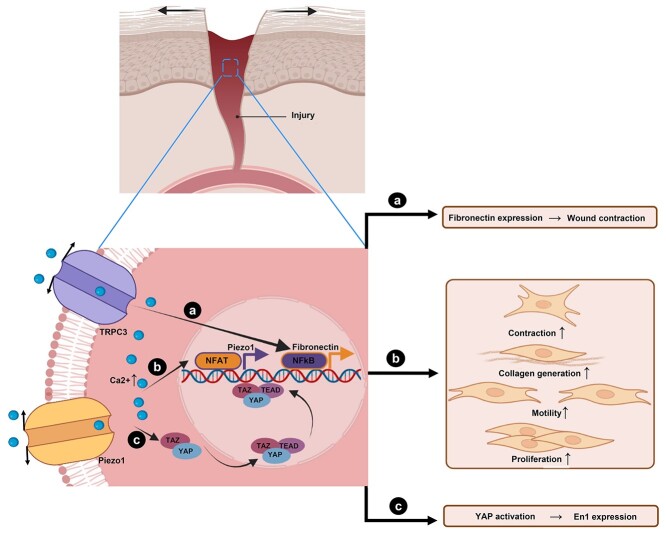
The schematic representation of the mechanical stretch-induced formation of hypertrophic scarring through transient receptor potential (TRP) C3 (TRPC3) (a); Piezo proteins like Piezo1 (b); and Yes-associated protein (YAP) (c). (**a**) TRPC3 channel plays a key role, as a force transducer, in the formation of hypertrophic scar. In response to mechanical stretch, the TRPC3 channel is activated and increases the calcium influx, which triggers nuclear factor-κB (NFκB) phosphorylation. The translocation of activated NFκB into the nucleus takes place subsequently and results in the expression of fibronectin and wound contraction [69]. (**b**) In hypertrophic scar, mechanical stretch localizes around the fibroblasts membrane, transferring from the matrix to Piezo1, and leading to Piezo1-mediated calcium influx. The Piezo1 activity is associated with enhancement of proliferation, collagen generation and differentiation in the presence of the force [74,75]. (**c**) Mechanical tension in the wound bed upregulates En1 expression, generating scar-producing En1 lineage–positive fibroblasts. YAP inhibition is related to the suppression of En1 activation in wounds [66]. *NFAT*: the nuclear factor of activated T cells. (Created with BioRender.com.)

Yes-associated protein (YAP) and PDZ-binding motif (TAZ) are transcriptional coactivators that commute between the cytoplasm and the nucleus in reaction to biomechanical signals. In the nucleus, YAP/TAZ proteins bind to the transcriptional enhanced associate domain (TEAD) family transcription factors and control the gene expression. Nuclear YAP/TAZ are responsible for enhancing cell proliferation, surviving stress, healing wounds and regenerating tissues. Intriguingly, YAP/TAZ senses a wide variety of mechanical cues and is involved in their translation into transcriptional programs [67]. It has also been shown that mechanotransduction through YAP primarily accounts for the conversion of wound En-1–negative fibroblasts to En-1–positive fibroblasts (Figure 2). Thus, the suppression of YAP is critical to inhibiting En-1–positive fibroblasts and eventually reducing scarring in a mouse model [66].

Fibronectin is a high molecular weight glycoprotein in the ECM and accumulates in fibrotic lesions [68]. Its expression in response to mechanical stretching leads to intractable hypertrophic scar contracture via the transient receptor potential (TRP) C3–nuclear factor-κB (NF-κB) axis [69]. TRP ion channels are a possible mechanical force transducer that seems to be highly expressed in human hypertrophic scar tissue. There is the accumulation of cytoplasmic calcium in TRPC3 overexpressing fibroblasts after repetitive stretching, which may be the result of NF-κB activation [70]. Additionally, Piezo proteins, i.e. Piezo1 and 2, are cation channels sensitive to mechanical loading [71]. As such, Piezo1 overexpression is observed in human and rat tissues with hypertrophic scars. Also, *in vitro* studies on cyclic stretching demonstrate elevation of Piezo1 level and Piezo1-mediated calcium concentration in human dermis fibroblasts. Leading effector proteins of calcium signaling are the nuclear factor of activated T cells (NFAT) family of transcriptional regulators [72]. An increase in intracellular calcium levels leads to the dephosphorylation of critical residues in NFAT proteins, facilitates NFAT nuclear localization, and induces target genes [73]. While the hypertrophic scar is being formed, mechanical force is transmitted from the ECM to Piezo1 localized at the cell membranes of dermal fibroblasts. This Piezo1 regulates cellular behavior by promoting cell proliferation, collagen generation, motility and contraction under stretching, resulting in the formation of hypertrophic scar [74,75]. Figure 2 shows how the application of mechanical stretch aggravates the formation of hypertrophic scarring via YAP activation and TRPC3- and Piezo1-mediated mechanisms. Together, inhibition of YAP, Piezo1 and TRPC3 might be therapeutic approaches to reduce scarring and direct regeneration.

Fibroblasts display direction-dependent alignment and elongation as exposed to mechanical stimuli. Applying tension to skin fibroblasts causes a remarkable change in the expression level of genes involved in ECM remodeling and inflammation [76]. High-tension wounds may be related to severe scarring, with forces being centered in the marginal part of keloids. A biomechanical bidirectional loading device induces hypertrophic scarring in the murine dorsal skin model [1].

The crosstalk between mechanistic factors, including ECM crosslinking and stiffening, mechanotransduction through regulation of integrin and focal adhesion kinase (FAK), and integrin-mediated TGF-β pathway, underlies tension-stimulated skin fibrogenesis [77]. The ECM-integrin-cytoskeleton signal pathway is responsible for controlling fibroblast actions, such as viability, collagen synthesis, and transformation into the myofibroblast. In this classical pathway, integrins are bidirectional machines that serve to sense mechanical stimuli and transduce information targeting cells and their ECM [78].

The ECM molecules, in association with the cytoskeleton, are involved in regulating cellular activity, shape, differentiation and migration [60]. Mechanical forces and inflammation could synergistically trigger Akt signaling, which, though not a critical hypertrophic scar pathway, involves potential upstream mediators like FAK [79]. Focal adhesion complexes like talin, vinculin and paxillin govern cellular interaction with ECM and stimulate FAK [80]. Together, integrin-FAK signaling mediates the transmission of mechanical signals to ECM and then the cell cytoskeleton. In the hypertrophic scar model, FAK can enhance the production of chemokine monocyte chemoattractant protein-1 by human dermal fibroblasts. The knockout of FAK in mice alleviates fibrosis development in addition to recruiting inflammatory cells [60].

The ECM stiffness can impact the mechanical signaling, triggers the expression of TGF-β1, the transition of fibroblasts to myofibroblasts, and affects the production of collagen. A stiff ECM activates integrins and then TGF-β1, which can bind to the TGF-β1 receptor present on myofibroblasts. Apart from increasing the levels of TGF-β1 and α-SMA, a positive feedback loop is formed that elevates the ECM stiffness and ultimately causes fibrogenesis. By contrast, a soft ECM is associated with low TGF-β1 production and decreased receptor binding, and low levels of α-SMA [77]. Targeting multiple pathways involved in mechanobiology is a new paradigm for the effective treatment to reduce cutaneous scarring.

### Mechanoregulation of the myofibroblast in wound healing and scarring

Activated fibroblasts or myofibroblasts that augment scarring and fibrosis can release high levels of ECM components with inappropriate structural and mechanical features. These ECM molecules have a changed potential to bind to the growth factors [62,63,81]. For instance, tenascin-C is an ECM component that consists of EGF-like repeats and is able to bind and activate the EGF receptor on the fibroblasts and keratinocytes membranes. This binding is of low affinity but high avidity [82]. If the ECM accumulation and crosslinking continue excessively, tissue stiffness and pathological scarring increase, which may end up in the impairment of skin function [62].

By adhesion to the ECM, activated myofibroblasts possess contractile forces that contribute to scar contracture. The integrin-FAK signaling pathway plays a key role in skin mechanotransduction. FAK is stimulated as a result of mechanical forces in the wound healing process, which subsequently impacts intracellular signaling through many downstream factors (e.g. phosphoinositide 3-kinase and mitogen-activated protein kinase), thereby mediating fibrotic responses [12,62,83]. While the downregulation of the FAK signaling pathway comes about in non-healing wounds, excessive FAK activation results in hypertrophic scars formation. Moreover, FAK degradation in wounds like diabetic ulcers contributes to delayed wound healing and abnormal scar formation [84]. Exposure of the wound area to the elevated tension gives rise to hypertrophic scarring [62,64]. Unlike postpartum wounds, there is lower resting stress in scarless fetal wound healing [12].

TGF-β1 has been used in many studies as a potential target to prevent pathological scars and is known to induce myofibroblast differentiation [85–87]. Besides TGF-β1, other factors, such as connective tissue growth factor, platelet-derived growth factor, insulin-like growth factor, vascular endothelial growth factor, and interleukin (IL) 6, are involved in the promotion of myofibroblast differentiation [64]. However, some secreted molecules, such as FGF, epidermal growth factor (EGF), interferon-gamma, and IL-10, exert inverse effects. Recently, several factors have been identified with a capacity for regulating myofibroblasts and, as a result, fibrosis; integrin α11β1, for example, mediates pro-fibrotic signals from fibroblasts in the dermis; collagen export from fibroblasts relies on thrombospondin-5; and integrin-linked kinase is a mechanotransducer with signal transmission activity, which can control TGF-β1 production [63]. The lack or suppression of these proteins *in vivo* could attenuate fibrosis and thus has been proposed as an interesting anti-fibrotic agent [63]. As scarring decreases with reducing mechanical loads on the injury site, scar treatment strategies can be directed at mechanical offloading [12,88]. Several biochemical signals correlated to mechanical tension are found to lower cutaneous scars, such as inhibition of TGF-β1, the addition of TGF-β3, or downregulation of connexin 43 [89]. A deep understanding of myofibroblast-centric mechanisms, particularly those involving mechanosensitive pathways such as integrin-FAK signaling, might result in a new therapeutic intervention to treat scarring.

### Keratinocytes, mechanical forces and wound healing processes

Mechanical forces play a key role in expanding skin progenitors and inflammatory cells in the wound site [90]. To recapitulate these mechanisms, the human keratinocytes and fibroblasts were seeded on a collagen membrane under tensile loading and exhibited an asymmetric migration of keratinocytes that is controlled by the release of EGFs from fibroblasts [91]. Three main mechanisms are underlying the tension-associate proliferation of keratinocytes: ECM-integrin signaling, mitogen-activated protein kinase-associated pathway, and epithelial-mesenchymal interactions [92]. Consequently, the migration and re-epithelialization of wound sites are dependent on integrin molecules and are necessary for wound closure [56].

In a study by Wong et al., the knockout of FAK in keratinocytes exerted an atrophic impact on the dermal layer. Such observations indicate how inter-cell-layer communications are complex in the mechanical signaling of multicellular tissues, such as skin. Upon mechanical stimuli, ECM-integrin is implicated in the upstream pathway that in turn triggers numerous downstream targets in epidermal cells. The mitogen-activated protein kinases, consisting of p38 kinase, can determine the biological response to wound tension [93]. The exposure of keratinocytes to pressure leads to the induction of p38 phosphorylation [91].

The keratinocyte to mesenchymal transition contributes to wound healing and fibrosis. Applying continuous tension to human epidermal cells enhances cellular proliferation along with their transition into mesenchymal lineages. The murine skin epidermal cells showed an epithelial-mesenchymal transition characteristic under mechanically stretched conditions [94]. Together, skin keratinocytes are key players in skin wound healing and scarring; thus, understanding and modulating their biomechanics allow the development of novel anti-scarring therapies.

### Other influential cells

The skin contains a complex microvascular network [95]. Throughout the development of skin tissue, vascular endothelial cells, akin to epidermal and dermal cells, receive mechanical forces, such as shear stresses. A body of studies has indicated that forces can enhance angiogenesis and vascular remodeling [96,97]. In particular, a rat ear stretch model revealed an increase in the population of epidermal cells and blood vessel density in response to continuous or cyclic tensions [98]. The intermittent exposure to external volume expansion at moderate intensity was reported to augment the density of the cutaneous vascular network and thickening of the entire skin in mice [99]. There is some evidence in support of high vessel density and dysfunction of blood vessels in hypertrophic and keloidal scars, thereby scars can be considered vascular diseases [100].

The subcutaneous adipose tissue is composed of different cell types, like adipose-derived stem cells, preadipocytes and mature adipocytes, and all are responsive to the biomechanical forces. Adipose-derived stem cells can differentiate into adipocyte lineage cells or vascular endothelial cells in response to appropriate mechanical stimuli [101]. Nevertheless, mature adipocytes contribute differently to adipogenesis upon cyclic stretch in comparison with preadipocytes; tension stimulates a signaling pathway in adipocytes, which leads to cell hypertrophy [102], while, on the contrary, such a pathway reduces in preadipocytes [103]. Skin is a multicellular tissue, triggering regeneration instead of scarring during wound healing requires crosstalk of many signaling pathways involving different skin cells, which finally offers important candidates for scar treatments.

### Leveraging the mechanical forces to prevent/minimize scarring

The compromised biomechanical function of scarred tissues necessitates new methods to recover the viscoelastic behavior of skin. These approaches are expected to enhance load transfer and strain compatibility between skin and scars, accordingly improving functionality along with aesthetics of the healed wound as stretch loading stimulates structural adaptation in both skin and scar tissues and realigns collagen rapidly in a parallel manner [104]. The therapeutic strategies based on the modification of scar tissue biomechanics are discussed as follows.

### Current clinical therapies

#### Silicone gel

According to the latest guidelines for scar treatment and prevention, silicone therapy, including silicone sheets and silicone gels, are the first-line treatment option for hypertrophic and keloidal scars [105]. The treatment of scars is based on occlusion and further hydration of scarred tissue [106]. Since the 1980s, silicone gel sheeting has been vastly applied in the clinic to treat hypertrophic scarring. The significantly improved outcomes were reported after gel sheeting; thus, they have been used as a standard practice in plastic surgery [107]. Although the cellular mechanism of silicone gel function is still understudied, silicone therapy provides external mechanical support whereby tension on the wound site decreases [18]. Mustoe et al. showed that occlusion and hydration suppress keratinocyte activation and also reduce fibroblast function [106]. Other studies reported a reduction in TGF-β1 and TGF-β2 expression levels in fibroblasts upon silicone therapy [108,109]. Akaishi et al. indicated the effects of silicone gel on the reduction of tensile stress in scars, recommending its application as a mechanomodulation tool [110]. Silicone gel can lower the tension along the border between scarred and unwounded skin tissues. The tension is transferred from the scar border to the silicone sheet lateral edge [110].

#### Pressure therapy

Applying compressive force by pressure garments was first introduced in 1860 for the treatment and management of hydrotropic scars in burn injuries [111]. During the 1960s, this treatment was considered the standard of care in burn wounds to speed up the remodeling stage of the wound healing process [111]. Prophylactic pressure is advised for skin grafting as well as burn injuries when the spontaneous closure of the wound does not occur within 10 to 14 days [112]. Pressure therapy takes advantage of compression bandages to transmit pressure to scarred skin, decreasing the perfusion pressure of capillaries as well as accelerating the maturation of collagen matrix that results in flattening the scar [113–115]. Additionally, the use of compression garments is suitable for full-thickness wounds, grafted wounds bordering a full-thickness wound, injuries to pediatric and young adult patients on the skin, lesions in those with dark complexions, and wounds in anatomic sites under compressive force [116].

Pressure can be applied to scars by massage that speeds up collagen maturation, controls scar tissue remodeling through the interruption of fibrotic tissue, promotes pliability, aids lysis and reorganizes the collagen matrix [117,118]. This is a commonly adopted treatment for flattening and softening the scars. A small amount of emollient is utilized to strongly massage the scar, and increase pressure to make it blanch without impairing the epidermis [119]. Since this treatment is not well supported with enough research data, the mechanisms of action of pressure therapy at the cellular and molecular levels remain unknown.

**Figure 3.**
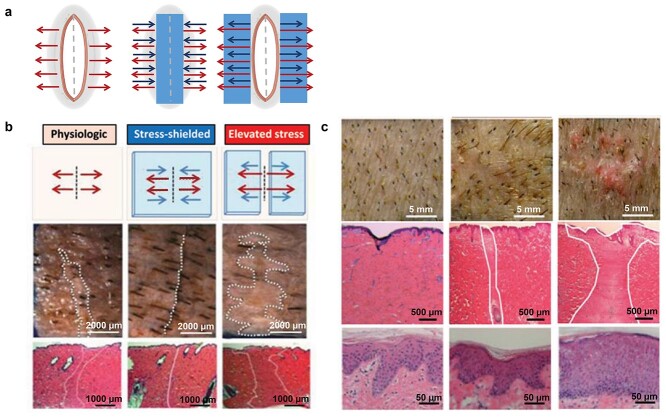
The effect of mechanical stress on scarring and skin regeneration in a pig wound model. (**a**) Schematic representation of wounds before and after applying the stress shielding device. The control wound is only under physiologic stress with no device. Then, the wound is shielded by directly placing the device (blue rectangle) over the wound. The red arrows indicate physiological skin stress and its direction. Conversely, the blue arrows represent the tension applied by the device and its direction. The highest stress level caused by para-positioned devices (which are shown by blue rectangles on either side of the long axis of the wound represented by a dotted line in the middle) results in maximal surface scarring, shown by longer red arrows. (**b**) Schematic representation of different stress states. The white dotted lines show scarring on the skin surface that is also determined in histological images by white lines. (**c**) Stress shielding significantly decreases cutaneous scarring in high-tension wounds. The unshielded high-tension wounds (right images) are found with considerable scarring and hypertrophy of the epithelial layer in the dermis. These observations resemble human hypertrophic scarring. Conversely, in the stress-shielded high-tension incisions (middle images), there is evidence of scarless wound healing with slight fibrosis and an epithelial layer similar to unwounded skin under physiologic stress (left images). Source: Adopted and reprinted with permission from [122]. Copyright 2021 Wolters Kluwer Health, Inc.

There are certain explanations based on hypoxia, biochemical changes and cell- and collagen-related effects. Indeed, pressure can regulate collagen production via restriction of blood flow, oxygen supply and nutrient availability in scars as well as promotion of the collagenase activity since pressure reduces the flow to the existing cells, thereby accelerating inflammation during healing [118,120]. While decreasing edema, pressure enables fluid to be forced out of cells. With the promotion of a hypoxic environment, fibroblasts undergo degeneration, and collagen release decreases [118]. Noteworthy, it attenuates collagen contents to the level observed in normal cutaneous scars and more quickly than the normal maturation process does; the realignment of collagen bundles is reoriented parallel as pressure forces the bundles into a certain direction [118,121].

### Emerging therapeutic approaches

#### Preclinical studies

Recent studies have dealt with biomechanical cues in the treatment of scarring [59,60]. One potential way before the use in a human would be the mechanical stimulation of these molecules *in vitro* or using animal models. Developing a topical device that manipulates mechanical forces during wound closure to reduce postsurgical scarring has been suggested. Januszyk et al. found upregulation of inflammatory and fibrotic pathways (i.e. FAK and extracellular signal-regulated kinase) in incisional wounds. They proposed the use of a biomechanical offloading approach to reverse the effects [55].

In a pig animal model, Gurtner et al. exposed cutaneous wounds to mechanical stress by employing a mechanomodulating polymeric device [122]. Stress shielding of skin incisions in a swine model diminished histologic scar area but elevated stress states that led to a dramatic decrease in profibrotic markers **(**Figure 3**)**. Wound closure under high tension resulted in scarring with a human-like phenotype successfully attenuated after stress shielding [122].

A rabbit ear model of hypertrophic scarring was used to compare silicone gel sheeting and paper tape. No difference was observed in scar elevation index based on histology and photographic analyses between these two treatments. Both treated wounds showed less *in vivo* scar formation photographically than untreated wounds [123].

**Table 1:** Mechanical modulation in different mechanotherapy techniques

**Mechanotherapy mode**	**Mechanical modulation at the injury site**	**Ref.**
Silicone gel	Reducing mechanical tension	[18]
Pressure therapy	Applying compressive forces	[111]
Stress-shielding	Recovering a balance between physiologic skin stress and external forces; Reducing mechanical tension	[122]
Microporous tape (Micropore™)	Reducing mechanical tension	[21,132]

**Figure 4.**
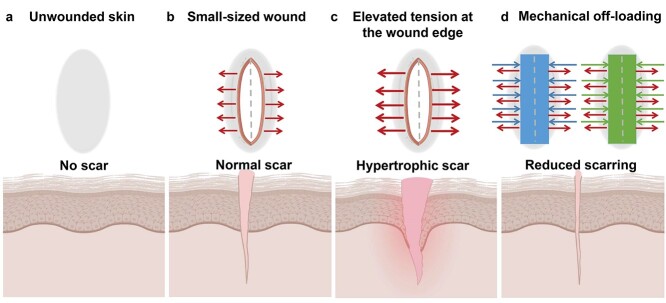
The presence of mechanical tension across skin wounds worsens scarring. Human skin always experiences tension. But, when an injury occurs, that extra tension causes the wound to spread wide apart and gradually form a scar. (**a**) The schematic representation of normal skin without scarring. (**b**) Small-sized wounds are subject to low tension and end up with insignificant scarring. The red arrows indicate low tension and its direction. (**c**) Severe wounds are associated with increased tension, particularly at the wound edge. The greater the tension in the wound, the greater the scarring, typically in the form of a hypertrophic scar. The longer red arrows represent elevated stress at the wound bed. (**d**) By contrast, mechanical offloading by either stress-shielding (left) or microporous tape (right) reduces scar formation. The red arrows indicate skin tension and its direction, whereas the blue and green arrows show tension generated by stress-shielding and microporous tape, respectively. (Created with BioRender.com.)

Although wound contraction is an important process during wound healing to keep wound edges together, extensive and fast wound contraction plays a pivotal role in the generation of mechanical stress and, ultimately, scar tissue formation [59,124,125]. To direct wound contraction and scarless healing, biomaterial-based approaches, referred to as contraction blockers, have attracted much attention. Contraction-blocking biomaterials control the orientation and assembly of cells and ECM fibrillar protein during wound healing [125]. Yannas et al. reported that collagen-glycosaminoglycan scaffolds could efficiently delay contraction [126]. More recently, our group has demonstrated that a biomimetic fibrous network is strong enough to withstand the wound contractile forces and enhance scarless healing [25]. The fibrous structure with a large surface area and high porosity supports gas exchange, nutrient uptake, targeted delivery and exudate absorption, thereby improving cellular migration and infiltration [127,128]. All in all, biomaterials trigger early regenerative responses in wound healing by imparting appropriate mechanical strength to the wounds along with reducing the contraction.

#### Clinical studies

There are two novel mechanical modalities in the clinic, namely adhesive microporous hypoallergenic paper tape, and dynamic stress shielding device, that offload tension (Table 1). The former is a core surgical principle that helps in wound closure under the minimum achievable tension to reduce wound problems. The effectiveness of this method is based on its preventative role in modulating and minimizing wound tension **(**Figure 4**)** since skin injuries in anatomical sites of high tension are more likely to develop hypertrophic scars [21,26,122,129–131]. In a human trial, the postoperative use of these tapes in women undergoing caesarian sections caused significant decreases in scar volume and occurrence of the hypertrophic scar over 12 weeks. However, one patient was found with a hypertrophic scar, with four developing stretched scars only after the tape removal. No significant adverse event occurred [21]. Rosengren et al. designed a single-blinded, controlled trial for a 12-week taping of sutured torso wounds. At six months of follow-up, overall scar appearance was remarkably better in taped individuals. Also, taping mitigated median scar width, with only one participant experiencing a worse than expected outcome [132].

Dynamic stress shielding attenuates tension across a wound likewise **(**Figure 4**)**. This is because of the load-bearing capacity of silicone sheets [122]. Applying dynamic stress shielding devices on humans in a phase I clinical study, Gurtner et al. reported that stress shielding of abdominal incisions improved scar appearance significantly [122]. Two randomized controlled trials show promising results in overall scar appearance [130,133]. A clinical trial of 65 healthy adults demonstrated that the device had significant improvements in overall appearance with no serious adverse events. This study indicated the first level 1 evidence of scar reduction in abdominoplasty incisions [134]. The device is now marketed under the trade name Embrace®.

According to the current preclinical studies, minimizing mechanical forces at the wound site can be considered a therapeutic target for optimal scar healing and skin regenerative rehabilitation. Additionally, the recent clinical trials provide evidence that tension reduction in the wound site by emerging mechanotherapies can fulfill the patient’s and clinician’s demands and reduce scarring. However, there are still challenging wounds susceptible to the development of keloids even in the absence of elevated tension. Furthermore, there is a lack of evidence supporting the application of these new therapies in case of severe scarring over large areas of the body seen in burn injuries. Therefore, future systematic and controlled studies should more precisely define mechanotherapies applicable to such challenging wounds and severe burn injuries.

We propose further investigations that either consider the combination of mechanical loading (pressure) therapies with tension offloading therapies for burn cases or develop more effective therapies based on physicomechanical and biomolecular cues for keloidal scarring. In this regard, the application of soft-robotic technologies with the transferring capability of quantifiable and consistent mechanical stimulus to the wound sites along with advanced screening technologies to identify and validate molecular and cellular interactions in response to the applied loads will pave the way for the safe use of skin mechanotherapy for patients with large non-healing wounds.

## Conclusions

The adult human skin has a limited capacity to repair upon wounding. In adult human skin, mechanical and chemical signals are interdependent and contribute to wound healing. The increased mechanical tension upon wounding enhances the cell expansion and ECM elongation, thereby changing the constituents of the skin’s mechanical environment and ultimately stiffening skin tissue and contributing to scarring. To overcome skin scarring, the reparative processes must be reverted or shifted toward proper scarless healing through manipulating skin composition and tension. The mechanotherapy modalities should be developed based on the mechanobiological properties of skin scars to overcome the existing clinical challenges. Moreover, utilizing the biomechanical offloading approach to reverse the biomechanical effects contributing to scarring has been proposed with promising results in human trials. Nevertheless, early intervention is a key in wound management; thus, mechanoregulation should be conducted during the healing process to avoid scar maturation. By using the contraction-blocking biomaterials during wound closure, we can also control the orientation and assembly of myofibroblasts and collagen fibers and have skin regeneration with optimal scar healing.

## Authors’ contributions

M. H.: Investigation, Data curation, Formal analysis, Visualization, Writing – original draft, Writing – review & editing. J. B.: Formal analysis, Writing – review & editing, Funding acquisition. K. K.: Formal analysis, Writing – review & editing, Funding acquisition. A. B.: Formal analysis, Writing – review & editing. A. S.: Conceptualization, Investigation, Data curation, Formal analysis, Writing – review & editing, Supervision, Project administration, Funding acquisition. All authors read and approved the final manuscript.
